# Home and community-based services coordination for homebound older adults in home-based primary care

**DOI:** 10.1186/s12877-018-0931-z

**Published:** 2018-10-11

**Authors:** Gregory J. Norman, Amy J. Wade, Andrea M. Morris, Jill C. Slaboda

**Affiliations:** West Health Institute, 10350 North Torrey Pines Road, La Jolla, CA 92037 USA

**Keywords:** Home-based primary care, Community-based services, Care coordination, Homebound

## Abstract

**Background:**

Medically complex vulnerable older adults often face social challenges that affect compliance with their medical care plans, and thus require home and community-based services (HCBS). This study describes how non-medical social needs of homebound older adults are assessed and addressed within home-based primary care (HBPC) practices, and to identify barriers to coordinating HCBS for patients.

**Methods:**

An online survey of members of the American Academy of Home Care Medicine (AAHCM) was conducted between March through November 2016 in the United States. A 56-item survey was developed to assess HBPC practice characteristics and how practices identify social needs and coordinate and evaluate HCBS. Data from 101 of the 150 surveys received were included in the analyses. Forty-four percent of respondents were physicians, 24% were nurse practitioners, and 32% were administrators or other HBPC team members.

**Results:**

Nearly all practices (98%) assessed patient social needs, with 78% conducting an assessment during the intake visit, and 88% providing ongoing periodic assessments. Seventy-four percent indicated ‘most’ or ‘all’ of their patients needed HCBS in the past 12 months. The most common needs were personal care (84%) and medication adherence (40%), and caregiver support (38%). Of the 86% of practices reporting they coordinate HCBS, 91% followed-up with patients, 84% assisted with applications, and 83% made service referrals. Fifty-seven percent reported that coordination was ‘difficult.’ The most common barriers to coordinating HCBS included cost to patient (65%), and eligibility requirements (63%). Four of the five most frequently reported barriers were associated with practices reporting it was ‘difficult’ or ‘very difficult’ to coordinate HCBS (OR from 2.49 to 3.94, *p*-values < .05).

**Conclusions:**

Despite the barriers to addressing non-medical social needs, most HBPC practices provided some level of coordination of HCBS for their high-need, high-cost homebound patients. More efforts are needed to implement and scale care model partnerships between medical and non-medical service providers within HBPC practices.

## Background

Approximately two million older adults aged 65 and older in the United States meet the criteria for being homebound [1, 2]. Homebound older adults have great difficulty living in their home independently, have high-levels of frailty and physical disability, and often have cognitive, behavioral, and psychiatric impairments [3, 4]. Without regular access to primary preventative care, they resort to high emergency department and hospital use as a way of coping with fluctuations in their physical health [5, 6]. Homebound older adults respond well to routine medical care provided in their home, known as home-based primary care (HBPC), because it is effective at keeping patients medically stable, preventing hospitalization, and reducing medical spending [5–9].

HBPC is a multidisciplinary team-based approach to providing longitudinal in-home medical care to high-need high-cost patients with limited mobility. It has been shown to be a care model that reduces costs per patient while maintaining quality of care as well as patient and provider satisfaction compared to usual care [10–12]. A major advantage of long-term care provided in the home is that it enables the physician to evaluate the patient’s home environment, and be responsive to changes in health status, patient goals, and family caregiving capacity [13]. Unfortunately, of the 2 to 4 million people in the U.S. who are homebound only about one quarter receives medical care at home [2].

Many HBPC practices in the U.S. are provider-led by a physician or nurse practitioner. The practice may also include a registered nurse, and medical assistants who support providers by triaging patients, assisting with patient intakes, and handling medication refills [11, 14]. Larger practices may employ administrative coordinators who provide scheduling, billing, procurement of supplies, and other administrative tasks; social workers who focus on the patient’s home environment and link patients to community supports and services; medical coders and billers; and transition nurses who facilitate the patient transfer from the hospital to the HBPC practice [3].

In addition to being medically complex, homebound older adults often have a variety of non-medical health-related social needs (also referred to as social determinants of health) that include: housing, transportation, nutrition, social support, and assistance with activities of daily living. There is evidence to suggest that unmet social needs significantly impact health outcomes, increase healthcare utilization and costs [15–18]. As a result, comprehensive HBPC must consider the full spectrum of patient needs, both medical and non-medical, to better support this complex population and enable homebound older adults to age in place [19–21]. Non-medical home and community-based services (HCBS) can allow older adults to remain in their homes and avoid long-term care facilities, a goal that is shared with HBPC providers and their patients [22]. Assessing and addressing these non-medical social needs requires coordination between medical providers and HCBS providers. However, there is a gap in understanding how HBPC practices coordinate with HCBS providers to meet patients’ unmet social needs. Specifically, more information is needed about how nonmedical services are requested, to what extent there is ongoing coordination with HCBS providers, and what factors are assessed related to patient eligibility for services.

Care management models have been developed that include coordination of medical and non-medical patient needs [5, 15, 19, 20, 23], but these models have not been widely adopted by primary care in the U.S [21]. These models typically include staff resources to conduct assessments, identify community resources/partners, and define communication pathways that can include electronic or verbal [15]. For example, a health plan and a local Area Agency on Aging (AAA) in Arizona partnered to assess for social determinants of health with high-risk, dually-enrolled Medicaid/Medicare patients living in their home and provided connections to the appropriate community services under AAA contract, conducted ongoing assessments, and shared information between AAA and the health plan [19].

Several barriers exist to connecting high-risk older adults with the HCBS services they need, many of which apply to HBPC practices. An estimated 70% of older adults in the U.S. will need HCBS at some point [24]. While many older adults want to learn about HCBS, they often lack knowledge of the available services in their area, how to secure them, or how to pay for them [24, 25]. Similarly, medical providers may not know or understand how to refer and coordinate HCBS services [25, 26]. Demand for HCBS services may be greater than the availability of services within the community [27]. Finally, a recognized significant barrier is paying for HCBS [5, 27]. In the U.S. HCBS are covered by state government Medicaid, however not all older adults who need services will meet Medicaid’s eligibility requirements, forcing individuals to pay from their personal funds [28]. Often, older adults must spend down their savings before they are eligible for Medicaid coverage [27].

Based on models such as the Chronic Care Model [29], which postulates effective care for patients with multiple chronic conditions requires systems of coordinated medical and social services, we aimed to better understand non-medical care coordination for HCBS within HBPC practices. We surveyed HBPC practices in the U.S. to learn the extent social needs of patients are assessed and coordinated, and to determine the most salient barriers HBPC providers encounter in the coordination process, and if those barriers impact the frequency of coordination for the practices. While this is mainly a descriptive study, we hypothesized barriers to non-medical care coordination would be positively associated with difficulty and frequency of coordination among HBPC providers.

## Methods

Development of the survey instrument was informed by reviewing the relevant literature and by a previous survey of members of the American Academy of Home Care Medicine (AAHCM) [3]. Additional questions were developed collaboratively through an iterative process with project team members. The final version of the survey contained 56 questions and was divided into four sections: 1.) Identifying and Assessing Non-Medical Social Needs (14 questions); 2.) Coordinating and Evaluating Home and Community-based Services (10 questions); 3.) Oral Health Care Needs (11 questions); and 4.) Practice Characteristics (21 questions). Oral health questions were not included in this analysis**.** Non-medical social needs were defined to include: transportation, home-delivered meals, food preparation, personal care (e.g., bathing, toileting, etc.), housekeeping, housing assistance, home modifications and/or repairs, caregiver supports and/or training, financial advice, legal advice, case management, and medication adherence.

A link to the online survey was posted in the AAHCM electronic newsletter and was available to all AAHCM members over an eight-month period (March to November 2016). The survey was administered using SurveyGizmo software (www.surveygizmo.com). Two reminders were sent to AAHCM members to encourage participation. Western Institutional Review Board reviewed and approved exemption status for this study.

### Analysis

The unit of analysis was the individual HBPC practice site. For multi-site house calls practices, respondents were asked to provide information about the specific practice site where they provided services. For cases where more than one response was received from a multi-site practice, responses were cross-checked by IP address and responder role within the practice to ensure that the response was not a duplication.

Data were analyzed using SAS Studio Release: 3.6 (SAS Institute Inc., Cary, NC, USA). Descriptive statistics were assessed for HBPC practice characteristics, including practice size and location, number of practice sites and settings, funding structure, and profit status. Descriptive statistics were also used to describe how HBPC practices addressed social needs, including whether HBPC providers assessed patients for social needs, and the extent they coordinated social needs as part of their HBPC practice model.

Using univariate logistic regression models, we analyzed associations between the five most common barriers to coordinating HCBS (availability of local service providers; eligibility requirements; insurance coverage; cost to patient; time delays) and three dependent variables: ‘Does your practice coordinate HCBS?’; ‘How difficult is it to coordinate HCBS?’; and of the practices that coordinate HCBS, ‘How often does the practice coordinate HCBS?’ (often/always vs. never, rarely, sometimes). A logistic regression model was also used to regress the three dependent variables on the number of barriers (0 to 5) to coordination reported.

## Results

### Survey respondents

A total of 150 responses to the survey were submitted online. Of the 150, eight surveys were not eligible for inclusion (respondents were not part of a HBPC practice). An additional 41 surveys were excluded due to insufficient data for analyses. The final analytic sample consisted of 101 surveys. All respondents were part of a HBPC practice located in the United States. Forty-four percent of respondents were physicians, 24% were nurse practitioners, and 32% were administrators or other HBPC team members.

### Practice characteristics

Nearly all practices (86%) reported that 75–100% of their patients were ages 65 and older, and 26 practices (26%) indicated their entire patient population was 65 or older. Table 1 presents characteristics of the HBPC. A majority of practices (58%) were in the Northeast or the Midwest regions of the U.S. and most practices operated in urban and suburban settings. Only 13% of practices operated in rural areas. This geographical distribution of practices is comparable to a previous survey of AAHCM members [3]. Practices tended (56%) to operate independently. Most practices (63%) reported a patient census of more than 500 and consisted of a single site (74%). Group practices (68%) predominated over solo practices (32%).

**Table 1:** Characteristics of Home-based Primary Care Practices

Survey Question	N	N%
What region of the United States is your practice located?		
Northeast	27	30%
Midwest	25	28%
Southwest	14	16%
Southeast	13	15%
West	12	14%
Primary sponsor/ owner of the practice?		
Independent Provider (MD, NP, PA) / Provider Group	50	56%
Hospital or Health System	22	24%
Other	7	8%
Home Health Care Company	4	4%
Government Organization	4	4%
Independent Investor Group	3	3%
Total practice census		
< 500 Patients	56	63%
≥ 500 Patients	33	37%
Number of sites that your practice operates		
1	74	74%
2+	26	26%
Predominant practice setting		
Urban	44	44%
Suburban/Rural	41	41%
≥ 2 Settings	16	16%
Practice Type		
Group or Other	61	68%
Solo	29	32%
Practice funding structure/revenue model *(Select all that apply)*		
Insurance Reimbursement (Medicare, Medicaid, private insurers)	78	77%
Self-pay	22	22%
Subsidized (hospital, health system, or philanthropy)	19	19%
Percentage of patients dually enrolled in both Medicare and Medicaid		
< 50%	61	74%
≥ 50%	21	26%
How are HCBS for your patients typically paid for?		
Medicaid	40	40%
Self-pay	20	20%
Profit status of HBPC your practice		
For profit	54	61%
Not-for-profit	34	39%
Does HBPC practice provide services for patients with primary Medicare Managed Care?		
Yes	69	78%
No	20	22%

Most practices (77%) reported receiving funding for HCBS through insurance reimbursement (Medicaid, Medicare Advantage); 22% reported self-pay patients; and 19% received subsidized funding from philanthropy or through a hospital or health system. Seventy-four percent of practices indicated that their dually enrolled (Medicare and Medicaid covered) patients constituted less than 50% of their practice. The most common methods of payment for HCBS was through Medicaid (40%) and self-payment from the patient (20%). Most of practices were for-profit (61%), and most practices (78%) provided services for patients with primary Medicare Managed Care.

### Assessing and addressing social needs

Table 2 presents practice operations around assessing patients’ non-medical social needs. Nearly all HBPC practices (98%) assessed patient needs for HCBS. Seventy-eight percent provide an assessment during the intake visit, 88% provide ongoing periodic assessments, and 88% document this in the patient care plan. Referrals for HCBS were typically initiated by the healthcare provider (64%) and caregivers (12%). Seventy-four percent of providers noted that ‘most’ or ‘all’ of their patients needed HCBS in the past 12 months. The most common service needs were personal care (84%) and medication adherence (40%).

**Table 2:** Practice Operations: Identifying and Assessing Social Needs

Survey Question	N	N%
Does your practice assess patient needs for HCBS?		
Yes	98	98%
No	1	1%
Unknown	1	1%
How are patient needs for HCBS assessed? *(Select all that apply)*		
Periodic ongoing reassessments	86	88%
Initial intake assessment	76	78%
Other	12	12%
Are HCBS needs documented in the care plan?		
Yes	86	88%
No	9	9%
Unknown	3	3%
What typically initiates a referral for HCBS?		
Healthcare provider recommendation / observation	63	64%
Caregiver request	12	12%
Social worker recommendation / observation	8	8%
Patient request	6	6%
Other	9	9%
How many of your patients or caregivers had HCBS needs (past 12 months)		
Most/All	73	74%
None/Few/Some	25	26%
What were the most common service needs? *(Select all that apply)* (Top 5 listed)		
Personal care (e.g., bathing, toileting, etc.)	82	84%
Medication adherence	39	40%
Caregiver supports / training	37	38%
Case management	35	36%
Transportation	31	32%

Table 3 describes current practices for coordinating and evaluating HCBS. Eighty-six percent of practices reported they coordinate HCBS for their patients, and of those who do, 85% do so ‘often’ or ‘always.’ Of these practices, the most common coordination activities were following up with patients (91%), assisting with applications (84%), and service referrals (83%). Forty-eight percent of practices had nurse practitioners provide HCBS coordination. Nearly all practices that coordinate HCBS connect with multiple service providers or agencies (92%). HCBS agencies were usually characterized as local community service agencies (72%), and individual HCBS providers (70%). Word of mouth from patients (44%) was the most common way of determining the quality of an HBCS provider. Only 8% assessed quality through other agencies such as AAAs, and only 1% used the internet.

**Table 3:** Practice Operations: Coordinating and Evaluating HCBS

Survey Question	N	N%
Does your practice coordinate HCBS for your patients?		
Yes	87	86%
No	14	14%
How often does the practice coordinate HCBS?^a^		
Often/Always	73	85%
Rarely/Sometimes	13	15%
What level of coordination is provided? *(Select all that apply)*^a^		
Follow up with patients and caregivers	79	91%
Assistance completing applications	73	84%
Make service referrals	72	83%
Determine eligibility for services	61	70%
Follow up with community service providers	55	63%
Identify services	46	53%
Assess service needs on an ongoing basis	34	39%
Who in the practice is responsible for coordinating HCBS needs for patients (or caregivers)? *(Select all that apply)*^a^		
Nurse Practitioner	41	48%
Physician	33	38%
Social Worker	25	29%
Case Manager	21	24%
Does the practice coordinate patient HCBS with one or more community service providers/agencies?^a^		
Yes, with more than one	79	92%
Yes, with one community service provider/agency	6	7%
No	1	2%
What types of organizations do you coordinate services with? *(Select all that apply)*^a^		
Local community service agencies (e.g., AAA, ADRC)	62	72%
Individual HCBS providers	60	70%
Hospital systems	45	52%
Senior centers	44	51%
When making a referral, how do you primarily determine the quality of HCBS providers/agencies?^a^		
Word of mouth from patient	38	44%
Other	18	21%
Report from service provider	16	19%
AAA, ADRC	7	8%
Internet	1	1%

Note. ^a^ Question asked only of respondents who replied, “Yes” to “Does your practice coordinate home and community-based services?”

Table 4 shows across all practices, 57% reported that coordination was ‘difficult’ or ‘very difficult.’ The most common barriers to coordinating HCBS included cost to patient (65%), and eligibility requirements (63%). When asked what would make coordination of services easier, no clear answer emerged. The top two answers included a point person in the practice to coordinate services for every patient (27%), and a local service that could handle everything (24%). Only 19% of practices reported having an EMR interoperable with HCBS providers.

**Table 4:** Barriers and Potential Solutions to Coordinating HCBS

Survey Question	N	N%
How difficult is it to coordinate HCBS for your patients?		
Difficult/Very Difficult	56	57%
Neutral/Easy/Very Easy	42	43%
Top barriers to coordinating HCBS for patients/caregivers *(Select all that apply)*		
Cost to patient	66	65%
Eligibility requirements	64	63%
Insurance coverage	61	60%
Availability of local service providers	40	40%
Time delays	40	40%
What do you think would make the coordination of HCBS easier? *(Select all that apply)*		
A point person in the practice to coordinate services for every patient	27	27%
A local service that could handle everything	24	24%
Other	16	16%
More knowledge of local available services	13	13%
Defined quality measures for long-term services and supports (LTSS)	12	12%
Unknown	9	9%
Is your practice EMR interoperable with other HCBS providers or agencies?		
No	66	81%
Yes	15	19%

### Barriers associated with HCBS coordination

We tested associations between the five most common barriers to coordinating HCBS (i.e., cost to patient; eligibility requirements; insurance coverage; availability of local service providers; and time delays) and whether practices coordinated HCBS; how difficult it was to coordinate services; and how often practices coordinate services (Table 5). Cost to patients was the barrier most strongly associated with practices reporting they conduct care coordination (OR = 2.96, 95% CI = 0.94–9.38, *p* = .06). Four of the five barriers were significantly associated with HBPC practices reporting it was ‘difficult’ or ‘very difficult’ to coordinate HCBS compared to those who reported it was ‘neutral,’ ‘easy,’ or ‘very easy’ (OR ranging from 2.49 to 3.94, all *p* values < .05). Number of reported barriers was also associated with an increased difficulty with HCBS coordination (OR = 1.77, 95%CI = 1.32–2.37; *p* = .0001). Frequently providing HCBS coordination was defined as those practices providing coordination ‘always’ or ‘often’ versus ‘sometimes,’ ‘rarely,’ or ‘never.’ Among practices indicating they currently coordinate HCBS services, patient eligibility (OR 5.96, 95% CI: 1.65, 21.55; *p* = 0.007) and time delays (OR 9.90 95% CI: 1.22, 80.11, *p* = 0.032) were associated with practices providing frequent coordination. When assessing the number of reported barriers, each additional barrier increased the odds that practices were frequently, rather than infrequently, providing coordination (OR = 1.88, 95% CI: 1.18, 2.99, *p* = 0.008).

**Table 5:** Associations Between Barriers to Coordination of HCBS and Coordination Difficulty and Frequency

	Does your practice coordinate HCBS?	How difficult is it to coordinate HCBS?	Of the practices that coordinate (*N* = 87), how often does the practice coordinate HCBS?
Barriers	OR, p-value	OR, p-value	OR, p-value
Provider availability	1.21, *p* = 0.75 For Yes: Barrier (87.5%) Not Barrier (85.3%)	*3.94, p = 0.003* For Difficult: Barrier (76.3%) Not Barrier (45.0%)	4.29, *p* = 0.07 For Often/Always: Barrier (94.1%) Not Barrier (78.6%)
Eligibility requirements	2.67, *p* = 0.09 Barrier (90.6%) Not Barrier (78.4%)	*2.49, p = 0.04* Barrier (65.1%) Not Barrier (42.9%)	*5.96, p = 0.007* Barrier (93.0%) Not Barrier (69.0%)
Insurance	2.29, *p* = 0.16 Barrier (90%) Not Barrier (80%)	*2.52, p = 0.03* Barrier (66.1%) Not Barrier (43.6%)	1.07, *p* = 0.92 Barrier (85.0%) Not Barrier (84.0%)
Cost to patient	2.96, *p* = 0.06 Barrier (90.9%) Not Barrier (77.1%)	*3.01, p = 0.01* Barrier (66.2%) Not Barrier (39.4%)	1.45, *p* = 0.55 Barrier (86.0%) Not Barrier (81.5%)
Time delay	1.21, p = 0.75 Barrier (87.5%) Not Barrier (85.3%)	*2.33, p = 0.05* Barrier (69.2%) Not Barrier (49.1%)	*9.9, p = 0.03* Barrier (97.1%) Not Barrier (76.9%)
Number of barriers	1.28, *p* = 0.15	*1.77, p = 0.0001*	*1.89, p = 0.008*

## Discussion

This study revealed that in the majority of HBPC practices, most or all their patients had nonmedical social needs within the past 12 months, and nearly all practices assessed these needs both initially and periodically on an ongoing basis. HBPC providers reported being actively engaged in assessing, documenting, and coordinating HCBS for their patients. Respondents indicated cost to the patient, eligibility requirements, and insurance coverage were the top barriers to coordinating HCBS.

The high patient need for HCBS reflects the patient population characterized as frail, medically complex homebound older adults, which is consistent with other reports on HBPC [14, 30]. Most of these patients are in the last years of life and their goal is often to remain at home and out of the hospital and long-term care facilities [22]. To meet this goal requires meeting the non-medical, social service needs of homebound patients. Medical care for these patients is mostly centered on keeping their chronic conditions stable and reconciling multiple medications.

Unfortunately, more than half of the providers indicated HCBS coordination was difficult or very difficult. While barriers did not differentiate if a practice coordinated HCBS, all the top five barriers were associated with HBPC practices indicating HCBS coordination was difficult. Eligibility requirements and time delays were the two barriers associated with frequency of HCBS coordination. This suggests that these two barriers may add to HBPC providers work load around HCBS coordination. The barriers HBPC providers most frequently reported their patients encountered were financial in nature, including the cost to the patient, eligibility requirements, and insurance coverage. This is consistent with other studies stating that in the U.S. Medicare coverage for HCBS is negligible, and those who need them often fall into a gap where they lack the financial means to pay for HCBS but do not meet Medicaid’s eligibility requirements to receive coverage [5, 27, 28]. Interestingly, while over three quarters of HBPC practices reported they provide medical services for Medicare Advantage (MA) patients, only about 12% of patients received HCBS through MA.

In the U.S., MA plans and other capitated payment models, as well as alternative payment models with shared risk or shared savings incentives, create opportunities to finance integrated medical and non-medical care for homebound frail older adults [15]. These models align Medicare payment incentives with conducting high-quality care to avoid high-cost medical care such as emergency department visits and hospitalizations [31]. For example, the Comprehensive Primary Care Plus Model (CPC+), an advanced primary care medical home model, is a proactive, team-based approach to care that focuses on a patient-centered care plan provided through home-visits, e-visits, and telephone [32]. The CPC+ model has several payment components including a care management fee, a performance-based incentive payment, and a fee-for-service payment. The model is specifically designed to address the longitudinal chronic care needs of medically complex older adults.

The other two top barriers to patients receiving HCBS services identified in the survey were availability of service providers and time delays. HCBS provider availability may reflect both lack of service options, and HBPC practitioners’ lack of awareness of the HCBS services available within their community [25]. Time delays and service availability both likely reflect scarce and stretched HCBS providers that may vary in quality of service delivery. Survey responses indicated there was no consistent method for HBPC practices to learn about and determine the quality of potential HCBS options. Patient word-of-mouth reports were cited as the top option providers relied upon to assess quality. A recent literature review concluded there is a need for quality measures to address the non-medical, health-related social needs of older adults [33]. Lynn [28] reaffirmed these needs, stating that most communities lack a centralized system for evaluating and determining the quality and parity of services, and reforms have failed to address these needs at a local level. Interestingly, 48 U.S. states do conduct quality assessments of HCBS in Medicaid as part of 1915(c) waivers, which allows states to shift care from institutional to community based settings [34]. However, these measures tend to be process-oriented and not focused on the quality of care beneficiaries receive. A quality measurement framework has been recently developed by the National Quality Forum to address gaps in performance measurement of HCBS in eleven domains (e.g., person-centered planning and coordination, community inclusion, and workforce) [35].

Practices endorsed several viable options to ease the burden of HCBS coordination. About a third of practices indicated a good option would be to designate a point person in the practice to manage this process. To address the implementation of possible HCBS coordination solutions, HBPC practices can look to existing innovative models of care that are partnerships between health care and community-based organizations that have been developed and evaluated [15, 36, 37]. Evidence supports the efficacy of nutritional assistance, case management and community outreach programs for high-need, low income older adults [38]. For example, the Geriatric Resources for Assessment and Care of Elders (GRACE) model was designed for low-income community dwelling older adults most of whom have multiple chronic conditions. The model includes in-home assessments and an individualized care plan developed by a nurse practitioner and social worker team in conjunction with the primary care physician. In a controlled trial over a two-year intervention period, GRACE reduced hospital costs for high-risk patients [23]. Unfortunately, only 10% of GRACE program costs are covered under traditional FFS Medicare, but the program would be feasible through Medicare managed care capitated payment [21]. While this program along with several other successful programs designed for high-risk older adults have common components such as comprehensive need assessments, patient-centered care planning, caregiver engagement, and care coordination, there has not been wide-spread adoption in practice [15, 36].

Study limitations include a low response rate to the survey and reliance on self-report measures, both of which may limit the generalizability of these findings. Of the approximately 1000 members of AAHCM, we received 101 complete responses. There may be a self-selection bias where HBPC providers who are already offering HCBS assessment and coordination in their practice were more likely to complete the survey. The proportion of HBPC practices that assess, refer, and coordinate HCBS may be lower in the overall population.

The study findings are from self-report survey responses, and there was no method to validate the responses. However, we have no reason to believe respondents had a ‘social desirability’ bias to exaggerate the extent of their care coordination efforts for patients. Other study methods, such as structured interviews, might result in information about barriers that did not surface through our survey. Future studies could use mixed-methods and include other stakeholders such as patients and family members to corroborate and expand on these findings. Study strengths include a comprehensive survey of non-medical care coordination completed by a diverse sample of HBPC practices in the US.

## Conclusions

Despite the barriers to coordinating HCBS, most HBPC practices in the U.S. provided some level of coordination of non-medical services for their high-need, high-cost homebound patients. More efforts are needed to implement and scale care model partnerships between medical and non-medical service providers for this vulnerable population of older adults, but adequate payment methods and quality measurements must be in place to ensure high quality care is delivered at a reasonable cost relative to other treatment options such as long-term institutional care and frequent inpatient care. Since ‘days spent at home,’ verses in health care facilities, has been recognized as the preference of most patients toward end-of-life [22], it makes sense to recognize ‘days spent at home’ as a patient-centered outcome that can be achieved through integrated care of HBPC and HCBS.

## Abbreviations

AAA: Area Agency on Aging
AAHCM: American Academy of Home Care Medicine
CI: Confidence interval
CPC +: Comprehensive Primary Care Plus Model.
FFS: Fee-for-service
GRACE: Geriatric Resources for Assessment and Care of Elders
HBPC: Home-based primary care
HCBS: Home and community-based services
MA: Medicare Advantage
OR: Odds ratio
